# Incidental finding of thyroglossal duct cyst in a neonate during endotracheal intubation: a case report

**DOI:** 10.1186/s12887-024-04742-x

**Published:** 2024-04-23

**Authors:** Emanuele Trovalusci, Carlo Pizzolon, Silvia Tesser, Stefano Doratiotto, Dalia Gobbi, Paola Midrio

**Affiliations:** 1 Pediatric Surgery Unit, Department of Women’s and Children’s Health, University of Padua, Padua, Italy; 2 Pediatric Surgery Unit, Ospedale Ca’ Foncello, Treviso, Italy; 3 Pediatric Anesthesiology Unit, Ospedale Ca’ Foncello, Treviso, Italy; 4 Radiology Unit, Ospedale Ca’ Foncello, Treviso, Italy

**Keywords:** Thyroglossal duct cyst, Sistrunk, Neonate, Midline neck mass

## Abstract

**Background:**

Thyroglossal Duct Cyst (TDC) is a common lesion of the midline neck, originating from an incomplete involution of the thyroglossal duct. It is typically observed in pre-scholar patients and surgery is the treatment of choice to prevent infections. Here reported a case of incidental diagnosis in a newborn patient.

**Case presentation:**

a 3-week-old male baby was admitted to our hospital for weight loss and projectile vomits after breastfeeding. After a diagnosis of hypertrophic pyloric stenosis, the baby underwent pyloromyotomy. During the endotracheal tube placement, the anesthetist noticed the presence of a midline neck mass. The suspect of TDC was confirmed by an intraoperative ultrasound, so, despite the age of the patient, we proceeded with the excision of the lesion according to Sistrunk’s procedure to avoid future complications and anesthesia.

**Conclusions:**

even if TDC is a common lesion of pediatric patients, anecdotical neonatal cases were described in the literature, all of them symptomatic. An accurate physical examination and ultrasound are essential diagnostic tools to distinguish TDC from other middle neck lesions, particularly ectopic thyroidal tissue. Sistrunk’s procedure is the most effective surgical approach. When diagnosis is made in a newborn, we suggest postponing surgery, unless the baby requires general anesthesia for other surgical procedures, such as in our case.

**Supplementary Information:**

The online version contains supplementary material available at 10.1186/s12887-024-04742-x.

## Background

Thyroglossal Duct Cyst (TDC) is the most common congenital lesion of the midline neck, affecting about 7% of the population. It is the result of an incomplete involution of the thyroglossal duct and, therefore, it can be found anywhere along the thyroid’s path of migration, from the foramen caecum to the sternal region [1].

TDC typically appears as an asymptomatic and circumscribed mass in the hyoid bone region filled with mucinous fluid, smooth and non-tender at the physical examination. Due to the continuity with the tongue, the cyst can get infected by oral bacteria. It may evolve into an abscess and eventually to intermittent drainage through a fistula opening on the skin or in the throat [2].

TDC is usually diagnosed in preschool-aged children, but it can be observed also in adult life, with a slight predominance for male patients [3]. A few cases of TDC in patients under 1 year of age are described in the literature, but there are no cases of accidental findings reported in newborns. Our experience with an incidental finding of TDC in a 3-week-old patient is hereby described.

## Case presentation

This was a male baby born at 40 weeks of gestational age by caesarian section after a failed labor induction, weighing 3450 g. Fetal ultrasonography (US) revealed left renal pelvis dilatation and borderline cerebral ventriculomegaly, but the baby was completely asymptomatic and discharged after birth. An US performed two weeks later confirmed the diagnosis of left hydronephrosis (IV grade), and dilatation of cerebral ventricles at the upper limit of normal ranges.

At 21 days of life the patient was taken to the emergency room for recurrent projectile vomits after each breastfeed and weight loss. US examination confirmed the suspect of hypertrophic pyloric stenosis, and the baby was admitted to the surgical ward and taken to the operating room the next day.

**Fig. 1:**
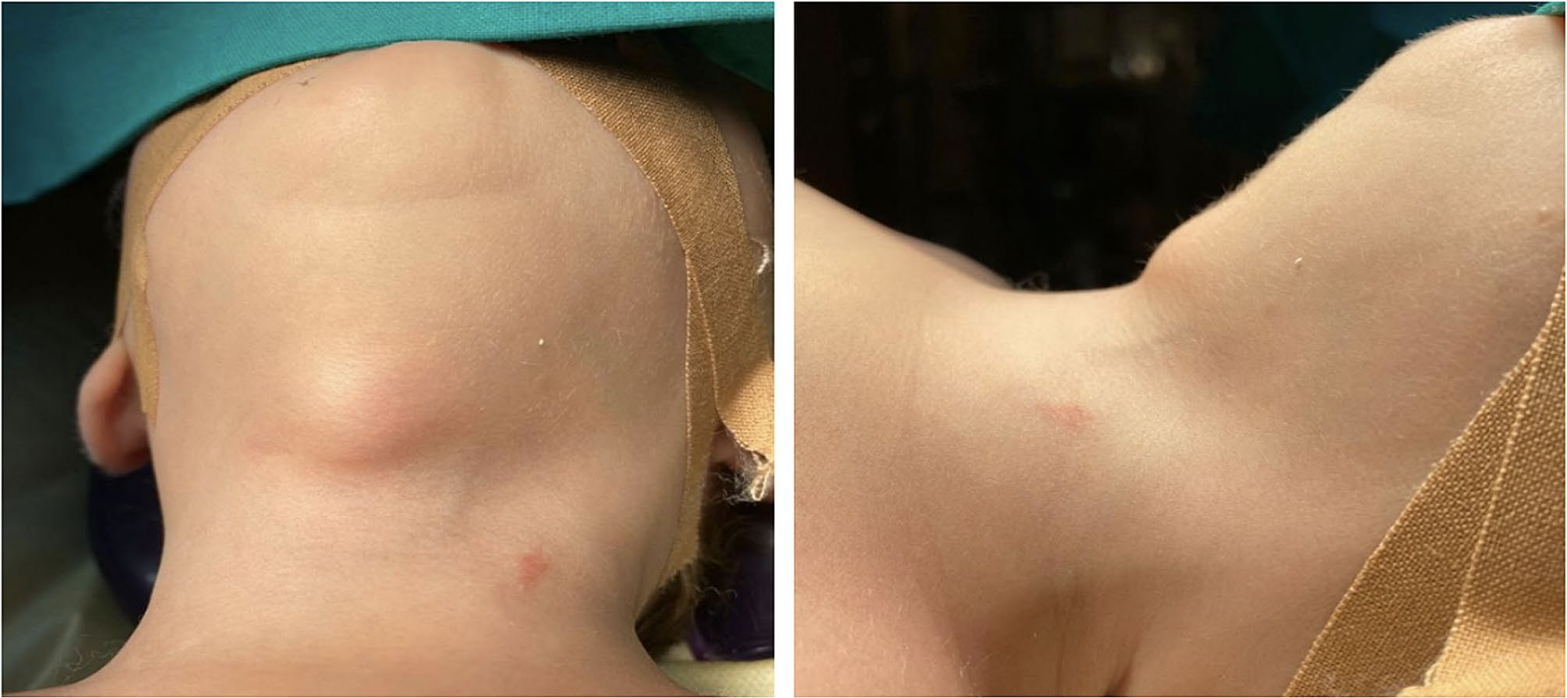
Pre-operative picture of the thyroglossal duct cyst after endotracheal intubation

During orotracheal intubation, the anesthetist perceived the presence of solid swelling on the midline of the neck (Fig. 1). An ultrasound was then performed in the operating room and revealed a 2 × 2 cm cyst filled with anechoic fluid and delimited by thin walls; the thyroid was normal. Despite the patient’s age, the finding was compatible with a TDC, and the decision was to proceed with the cyst removal, after the pyloromyotomy, to prevent future episodes of inflection and to avoid another surgical session. After an anesthesiologist and surgical team discussion, an open pyloromyotomy was performed with a small supraumbilical incision according to Bianchi. The procedure was completed without issues and the abdominal wall closed in layer with absorbable sutures.

**Fig. 2:**
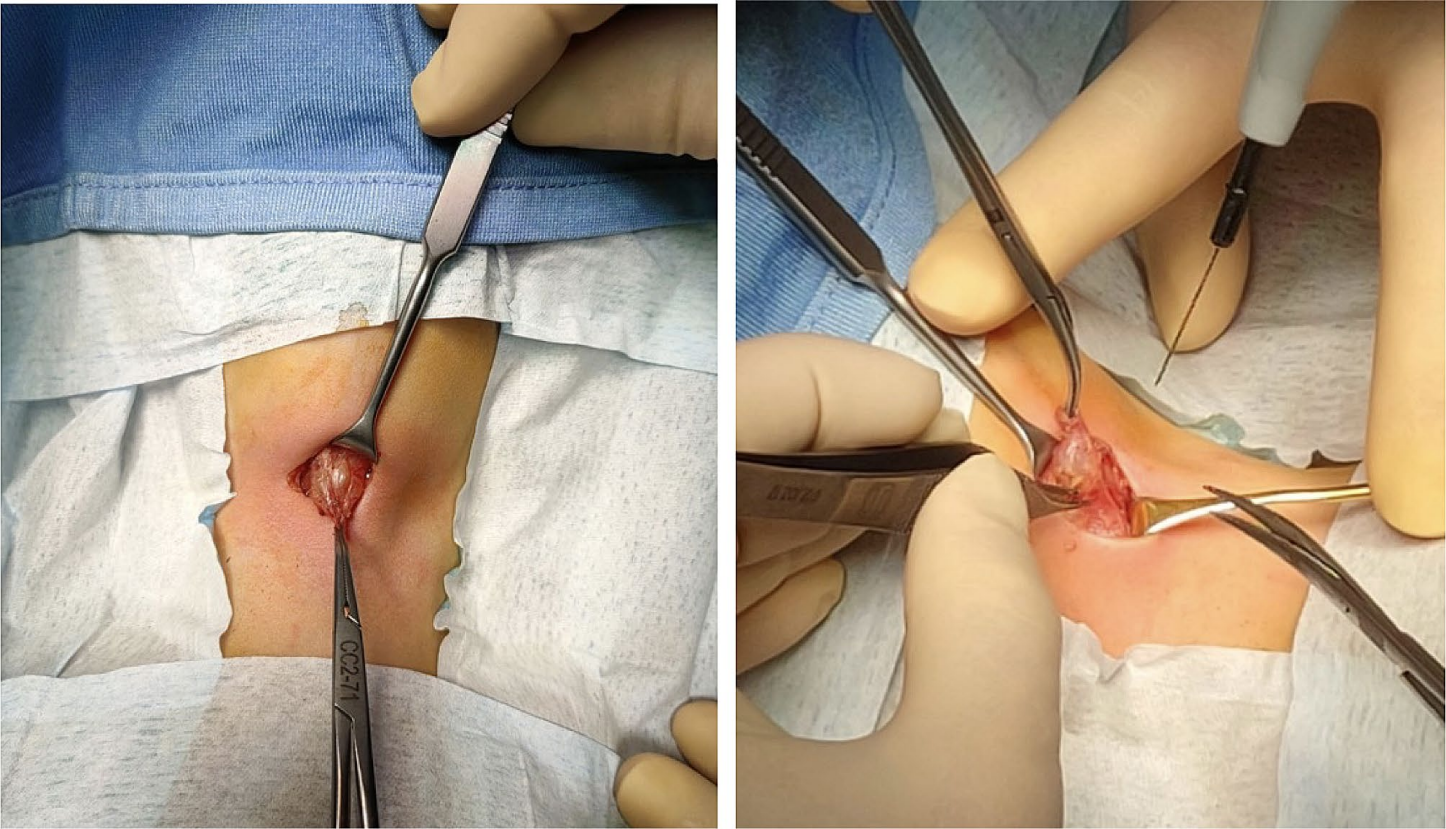
Thyroglossal duct cyst isolation during Sistrunk’s procedure

Upon the parents’ agreement, the cyst was removed according to Sistrunk’s procedure (Fig. 2). Considering the age of the patient, a 2.5X optical magnification was used to perform the anatomical dissection of the thyroglossal duct and cyst, plus the middle portion of the hyoid bone dissection, to avoid any damage of the respiratory tract, vessels, and nerves. An abundant presence of colloid material was observed during the cyst isolation, reinforcing the diagnostic suspect.

The postoperative course was regular. Analgesia was obtained with 15 mg/kg of paracetamol three times per day and a progression feed started the day after with 15 ml of formula milk *per os*. The child reached the full feeds on the same day, and no episodes of vomiting were documented. The baby was discharged on the 3rd postoperative day.

Histopathologic examination of the cyst confirmed the diagnosis of TDC. A genetic consult was also requested considering the presence of multiple anomalies, but the family refused to perform further analysis.

## Discussion and conclusions

TDC should always be considered when evaluating a midline neck mass in a pediatric patient. The usual presentation consists of an asymptomatic mass which can sometimes be detected by parents. The cyst can also become evident after infections, appearing as a swollen and painful mass. If not treated, the infection can lead to spontaneous rupture and evolve into a draining sinus. This occurrence is more frequently associated with an upper respiratory infection [2].

In our case, the features and position of the lesion resembled an asymptomatic TDC, even if the age of the patient was atypical. Different studies disagree on the mean age of presentation for TDC. In fact, TDC has a bimodal age distribution, with a peak in the first and the fifth decade of life, being frequently observed in pre-scholar age children [4]. Anecdotic cases of TDC diagnosed in infancy have been reported in the literature, and the earliest presentation was observed in a 3-month-old baby [5].

Many differential diagnoses should be considered when a midline neck lesion is observed in a pediatric patient, in particular dermoid cysts, pilomatrixomas, branchial cleft remnants, lymphadenopathy, and ectopic thyroid [2]. A careful physical examination, asking the patient to extend the neck and swallow or protrude the tongue, could be helpful to distinguish TDC from other lesions of the neck [6]. Indeed, TDC usually moves accordingly to the tongue due to its attachment to the hyoid bone. This explains why the anesthetist was able to identify the cyst in our patient during the endotracheal tube placement.

To confirm our suspicion and exclude thyroid anomalies, an US was performed at the operating table. TDC usually appears as a thin-walled and well-circumscribed anechoic/hypoechoic cystic lesion, strictly associated with the hyoid bone [7]. Sometimes cysts could be filled with debris secreted by the epithelial cells, especially after episodes of infection or inflammation [8]. US imaging is also mandatory to exclude the presence of median ectopic thyroidal tissue in or near the thyroglossal duct. If this is the case, thyroid hormones should be dosed before surgery, because in 75% of cases, the ectopic thyroid is the only functional tissue [8]. Second-level imaging exams, such as CT, MRI, and thyroid scan, could be helpful in inconclusive cases.

US revealed that the midline lesion of this patient had the typical characteristic of a TDC and the thyroid was normal. For this reason and the concomitant general anesthesia, we decided to proceed with TDC excision after pyloromyotomy. Surgical management is the best therapy in the case of TDC and it should be performed early to avoid infections or malignant degeneration (about 1% of cases) [4]. Sistrunk’s procedure represents the gold standard treatment because it has the best outcome in terms of recurrence and complications [9]. It consists of the excision of the cyst together with the central portion of the hyoid bone and the thyroglossal duct tract connected to the base of the tongue. A wide core of surrounding tissues should be excised to remove all the remnants that could cause recurrence [10, 11].

Our patient presented a series of pathological conditions, such as high-grade hydronephrosis, borderline ventriculomegaly, hypertrophic pyloric stenosis, and TDC, and a genetic consultation was requested. No specific syndromes were identified, and the CGH-array exam was suggested but was refused by the parents.

In conclusion, even if extremely rare, the diagnosis of TDC should be considered when a midline neck lesion is observed in a newborn baby. In our opinion, the excision of the cyst should be performed to avoid infections and malignant degeneration; however, we also recommend evaluating the risks and benefits of the procedure in babies < 6 months, considering the impact of general anesthesia neurodevelopment of these patients, but also the availability of proper neonatal surgery facilities to guarantee an uneventful surgical and postoperative outcome in such small patients.

### Electronic supplementary material

Below is the link to the electronic supplementary material.

Supplementary Material 1

## Data Availability

The authors confirm that the data supporting the findings of this study are available within the article.
