# Supplementary material for: Incidental finding of thyroglossal duct cyst in a neonate during endotracheal intubation: a case report
Source: BMC Pediatr. 2024 Apr 23;24:264. doi: 10.1186/s12887-024-04742-x (PMC11040807; doi:10.1186/s12887-024-04742-x)
Supplement: Supplementary file 1 — Supplementary Material 1 [file 12887_2024_4742_MOESM1_ESM.docx]

**
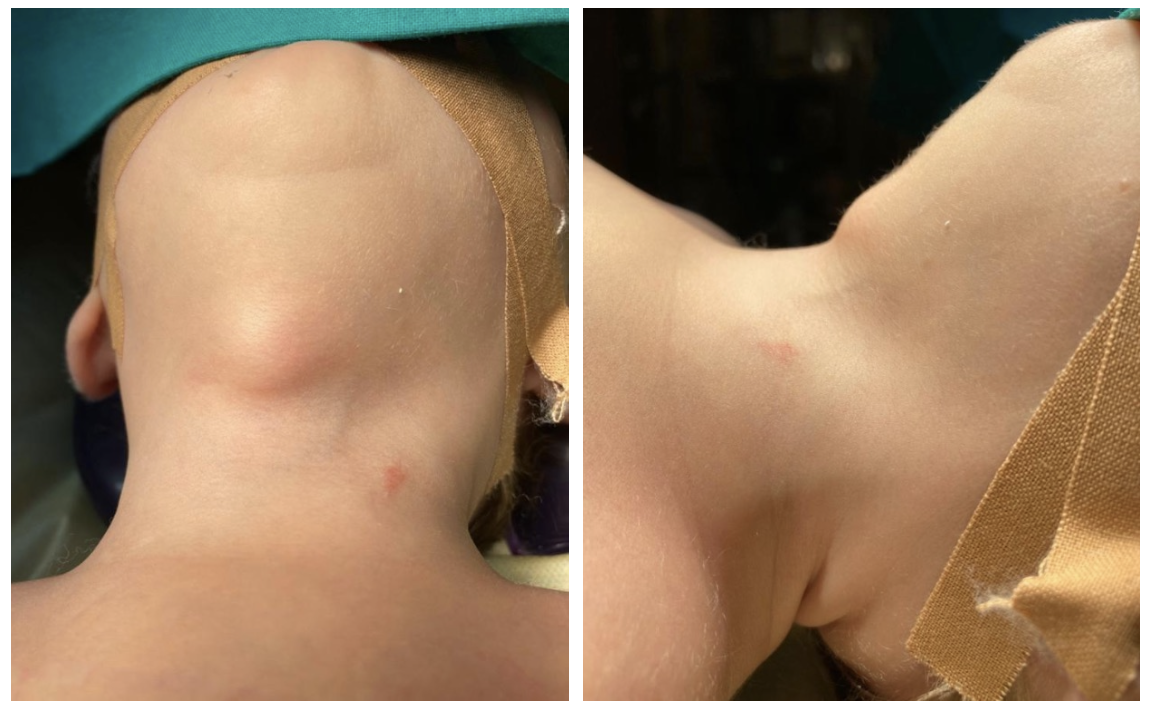
**

Thyroglossal Duct Cyst (TDC) is a common lesion of the midline neck, originating from an incomplete involution of the thyroglossal duct. It is typically observed in pre-scholar patients and surgery is the treatment of choice to prevent infections. No neonatal cases were ever described in a literature. Here reported a case of incidental diagnosis in a 3-weeks old male baby admitted for hypertrophic pyloric stenosis.
